# Path Planning of a Mobile Delivery Robot Operating in a Multi-Story Building Based on a Predefined Navigation Tree

**DOI:** 10.3390/s23218795

**Published:** 2023-10-28

**Authors:** Jordi Palacín, Elena Rubies, Ricard Bitriá, Eduard Clotet

**Affiliations:** Robotics Laboratory, Universitat de Lleida, Jaume II, 69, 25001 Lleida, Spainricard.bitria@udl.cat (R.B.);

**Keywords:** mobile robot, path planning, Dijkstra’s algorithm, package delivery, multi-story building

## Abstract

Planning the path of a mobile robot that must transport and deliver small packages inside a multi-story building is a problem that requires a combination of spatial and operational information, such as the location of origin and destination points and how to interact with elevators. This paper presents a solution to this problem, which has been formulated under the following assumptions: (1) the map of the building’s floors is available; (2) the position of all origin and destination points is known; (3) the mobile robot has sensors to self-localize on the floors; (4) the building is equipped with remotely controlled elevators; and (5) all doors expected in a delivery route will be open. We start by defining a static navigation tree describing the weighted paths in a multi-story building. We then proceed to describe how this navigation tree can be used to plan the route of a mobile robot and estimate the total length of any delivery route using Dijkstra’s algorithm. Finally, we show simulated routing results that demonstrate the effectiveness of this proposal when applied to an autonomous delivery robot operating in a multi-story building.

## 1. Introduction

In the last few years, e-commerce has greatly proliferated worldwide. From 2021 to 2022, e-commerce grew 6.5%, representing 19% of all retail sales [1]. This trend is associated with a growth in package deliveries [2,3], which adds complexity to city logistics and the tasks developed by parcel service providers [4]. Last mile delivery is a research area that deals with these topics in which all the logistical operations between depots and consignees are studied [5]. The main challenges related to last mile delivery include costs, time pressure, sustainability, increasing volumes and an aging workforce [6]. For example, delivery vehicles operate inefficiently, travel unnecessary distances [7], cause almost 20% of city congestion and emit up to 60% of total emissions [8]. As stated by Anderluh et al. [9], modern city logistics must evolve to create a more efficient model with minimal negative externalities. Conventional delivery has proven to be inadequate for the future [10], so current lines of research are assessing alternative package delivery concepts to identify efficient delivery systems for urban areas [11,12].

The transportation and delivery of packages are repetitive actions that can be automated with different types of automatic vehicles and robots [13]. An autonomous mobile robot is advantageous in that its computational power can be used to autonomously plan its trajectory, directly interact with other machines and dynamically react to changes in unstructured environments [14].

Outdoor last mile delivery with mobile robots is a practical problem that has caught the attention of many researchers [13,15,16,17,18], but the specific problem of indoor delivery in large buildings has been less studied. Outdoor last mile delivery is related to the transportation of packages between depots and customers’ residences, which are usually single-family houses, while indoor last mile delivery can be defined as the transportation of packages from the hall of a multi-story building to a specific apartment or office. Currently, the conclusions obtained in outdoor last mile delivery have been expanded and applied in indoor delivery applications. For example, Hutter et al. [19] proposed a four-legged robot that can pass through stairways and deliver packages in front of a door, and Castillo et al. [20] proposed the application of reinforcement learning to define a robust feedback motion policy for a two-legged humanoid robot that can be used for small-package delivery. Even though outdoor and indoor last mile delivery share denomination, the requirements for the systems involved in each method are different.

In an indoor and multi-story application, an autonomous delivery robot requires the implementation of a sequence of high-level actions to complete the tasks of transporting and delivering packages from a known pick-up point to a known drop-off target destination [21]. The planning of the trajectory of a mobile robot [22,23] requires an optimized procedure combining the spatial and operational information of the environment. The spatial information involves the position of all possible destinations as well as the navigable areas available to reach these destinations [24,25] and allows the use of a graph search algorithm to obtain an optimal path [26]. The operational information involves the procedures to navigate in the building such as interacting with elevators [27,28,29,30,31] and doors [32,33,34,35,36]. The spatial information is usually referenced in a 2D map [37,38] which describes the metric, topology, navigable areas, and appearance of the application scenario [39,40,41,42]. Additionally, the control of the motion of a mobile robot also requires a local self-localization procedure to properly follow the planned path [43,44,45,46,47], because the global positioning system (GPS) used in outdoor applications is inoperable indoors [48,49]. In indoor mobile robots, the common methods used to provide a periodic estimation of the absolute position of the robot in a map are commonly based on vision [50,51] and light detection and ranging (LiDAR) [52,53]. The information provided by LiDAR devices can be processed using different approaches, such as by using an iterative closest point (ICP) algorithm [54,55] matching raw LiDAR data [56,57,58,59,60,61,62,63], preprocessing the point clouds to detect geometric features before the matching [64,65,66,67,68,69], or using the raw LiDAR information as the input data for feature matching based on deep learning methods [70,71,72,73,74,75,76,77]. Finally, the routing strategy requires an estimation of the position of the mobile robot which follows the planned path [78,79] until arriving at the destination.

In the scientific literature, indoor navigation in multi-story environments has been addressed using different approaches. Kang et al. [80] proposed an algorithm to make a robot to get in an elevator to move to a goal floor. Their proposal was based on a mobile robot that was able to detect the floor number, but it was not able to push the panel buttons, so additional help was needed for it to interact with the elevator. Van Toll et al. [81] proposed an algorithm to automatically create a navigation mesh to generate visually convincing paths for multi-layered environments. In their proposal, the navigation mesh was represented by a collection of two-dimensional polygons defined in a single plane and with a set of connections. Zhang et al. [82] proposed a hierarchical path planner for autonomous robot navigation in multi-story buildings. In their proposal, the planner uses a topological graph with two kinds of nodes integrating the topological and metrical information of the environment to allow mobile robot navigation in the building. Liu et al. [83] proposed an ant colony optimization algorithm for multi-story building animation. Their proposal was claimed to be suboptimal in cases in which no previous navigation information was available. Joo et al. [84] proposed a semantic navigation framework to describe multi-floor environments connected by elevators. In this case, a semantic information processing module identifies which floor the robot is on from labels and other characteristic furniture objects. Li [85] discussed the stochastic path problem in an uncertain and chaotic world for which no previous navigation information is available. This approach involved a Markov decision process to find the best path in a multi-floor grid world affected by chaotic disturbances. Yuang et al. [86] addressed the problem of integrating both indoor and outdoor path-planning algorithms for mobile robot navigation in a multi-story building. Their proposal combined indoor and outdoor paths by the definition of specific entrance and exit nodes.

In general, an autonomous mobile robot uses a routing strategy that combines path planning and motion planning [87] because the presence of obstacles in the mobile robot’s trajectory may require an update of the current planned path [14,26]. In this work, the spatial and operational information required to plan the path of a mobile robot is represented as a weighted graph that defines the navigation tree of a target multi-story building. The proposed navigation tree has two particularities. Firstly, the nodes of the graph define the physical locations of the pick-up and drop-off points and some intermediate maneuvering points. Secondly, the segments of the graph define the straight collision-free paths between nodes. This proposal of using only straight trajectories between nodes requires the definition of intermediate navigation nodes in the navigation tree of the building.

In the scientific literature, there are different graph search algorithms that can explore a graph or navigation tree to find the shortest path between two nodes. There are optimization algorithms that are guaranteed to find the shortest path [88,89,90] and heuristic algorithms are not guaranteed to find the shortest path [91,92]. Examples of optimization algorithms are as follows: Dijkstra’s [88] algorithm, which solves the single-source shortest path problem from a single-source node to all the nodes of a graph; the Floyd [89] algorithm, which solves the shortest paths between all pairs of nodes in a graph; and the A-star [90] algorithm, which is an extension of Dijkstra’s algorithm [88] and the best-first search [93]. The A-star [90] algorithm uses admissible heuristics [90] to solve the single-source shortest path problem from a single node to a destination node. The main advantage of the A-star search is that there is no other optimization algorithm guaranteed to expand fewer nodes, so it is intensively applied to explore dense or massive graphs in complex problems [90]. Alternatively, Dijkstra’s algorithm is optimal to explore a sparse graph because it guarantees the exploration of all visitable nodes [88]. Finally, examples of heuristic algorithms used in path planning are genetic algorithms [91,94,95,96] and simulated annealing algorithms [92,97,98,99].

### 1.1. Problem Definition

In summary, the problem addressed in this paper is planning the path of a wheeled mobile robot that must transport and deliver small packages inside a multi-story building, navigating between floors using the elevators. To solve this problem, it is necessary to combine spatial and operational information, such as the location of the origin and destination points and how to interact with elevators.

### 1.2. Proposed Solution

This paper proposes a way to plan the path of a mobile delivery robot in a multi-story building based on the use of a manually created weighted navigation system that uses all feasible origin and delivery points as nodes. This weighted navigation tree and the Euclidean distances between the nodes are combined to define a small weighted sparse graph which is explored using Dijkstra’s [88] algorithm to find the shortest path between the origin and destination nodes.

Once a path has been found, the routing algorithm of the mobile robot follows the path. In case of the detection of an obstacle blocking the path, the graph must be updated [14,26] to discard the segments and nodes that are blocked and then explore again to search for the new shortest path to the destination node.

The main contributions of this work are as follows:

- The manual definition of a navigation tree describing the spatial information required to autonomously move in a multi-story building, using the elevators as connectors between floors.
- The description of how a graph created from this navigation tree can be explored using Dijkstra’s [88] algorithm to obtain the shortest path from a starting point to a destination point.
- The formulation of a distance–task matrix that can be used to estimate the total length of the trajectory of a mobile delivery robot moving in a multi-story building.
- The presentation of simulation examples that demonstrate the effectiveness of this proposal in the case of a mobile robot designed to transport and deliver small packages in a multi-story building.

This work is inspired by the contribution of Kim et al. [100] and is a continuation of the work of Palacín et al. [31]. Kim et al. [100] proposed a method to estimate the travel time of an indoor delivery robot working in a building with an elevator. Kim et al. [100] planned the path of a robot by the application of a genetic algorithm based on a specific heuristic. The analysis of the paths obtained suggested the definition of a navigation tree to reduce the complexity of the stationary delivery problem and avoid the use of genetic algorithms. Complementarily, in a previous work [31], we demonstrated that a mobile robot using 2D LiDAR and ICP matching for self-localization could take a remotely controlled elevator and navigate between floors, so this proposal is used for floor navigation.

### 1.3. Assumptions and Limitations

The assumptions made in this work to solve the path-planning delivery problem are as follows:

- A detailed 2D point cloud map of the multi-story building is available [31].
- All locations where a package can be picked up or dropped off inside the multi-story building have been identified as nodes and do not change during transportation.
- The multi-story building has remotely controlled elevators that can be directly accessed by a mobile robot via a wireless communication protocol [30].
- A navigation tree for each floor of the building has been manually defined.
- A segment (connection, link or vertex) defined in the navigation tree depicts a straight collision-free trajectory between two nodes. This straight trajectory can be blocked by the presence of dynamic obstacles.
- Packages are only delivered to locations that have their doors open. The problem of opening and closing the doors of the rooms is not covered in this work.

### 1.4. Structure of the Paper

The paper is structured as follows. Section 2 introduces the materials and methods used: the reference mobile robot, the reference map, the navigation tree and Dijkstra’s algorithm. Section 3 defines the implementation steps of the path-planning and motion-planning procedures, detailing the problem-solving method. Section 4 provides an overview of the simulation results obtained and the basic parameter settings used in the simulations. Section 5 analyzes and discusses the results obtained from the simulation, drawing conclusions and outlining future research directions.

## 2. Materials and Methods

The materials and methods used in this work are the model of the mobile robot that will be used in future works as a delivery robot, the 2D point cloud map of a real multi-story building, the navigation tree used to represent the spatial and operational information required to navigate between floors and the graph search algorithm used to plan the path of the mobile robot. 

### 2.1. Model of the Reference Mobile Robot

The mobile robot used as a reference for the simulation of the routing strategy is the APR-02 mobile robot prototype developed by the Robotics Laboratory at the University of Lleida (Spain) [101]. The APR-02 prototype is a human-sized (1.76 m, 30 kg) omnidirectional mobile robot with a motion system based on three omnidirectional wheels [102]. This versatile mobile robot has been assessed when performing tasks such as being a walking assistant [103] or using its forearms as a walking support [104]. In future works, the APR-02 mobile robot will be used as multi-story autonomous delivery robot.

**Figure 1:**
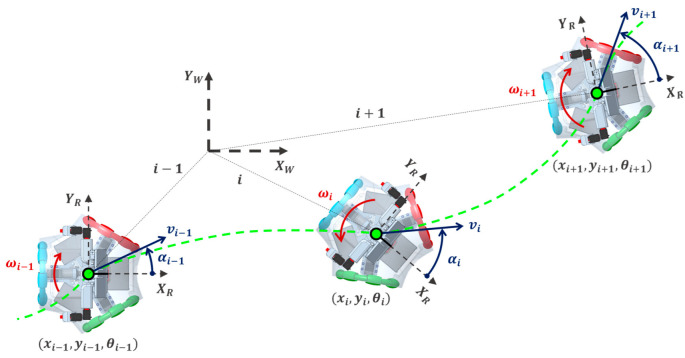
Parametric definition of the omnidirectional motion system of the APR-02 mobile robot. ($X_{R},Y_{R}$) represents the mobile robot frame in which $X_{R}$ is the front of the mobile robot. The parameters ($v_{i},\alpha_{i},\omega_{i}$) are the instantaneous motion of the robot, represented with blue and red arrows, while the green dotted line represents the trajectory.

The use of an omnidirectional mobile robot to transport and deliver packages is advantageous in that it can move in any direction and rotate without performing any maneuver [105,106,107,108], a motion that cannot be replicated with differential drive mobile robots [109,110,111]. The APR-02 mobile robot is able to execute any instantaneous motion $M_{i}=(v_{i},\alpha_{i},\omega_{i})$ defined in the robot frame $\left(X_{R},Y_{R}\right)$, where v is the module of the target lineal velocity of the robot, α is the angle of the lineal velocity relative to $X_{R}$ and ω is the target angular velocity of the robot. Figure 1 represents the parametric definition of the omnidirectional motion system of the APR-02 mobile robot. The instantaneous position of the mobile robot in the world frame $P_{i}=(x_{i},y_{i},\theta_{i})$ is computed using the following:

(1) $${\left[\begin{array}{c} x_{i}\\ y_{i}\\ \theta_{i} \end{array}\right]}_{World}={\left[\begin{array}{c} x_{i-1}\\ y_{i-1}\\ \theta_{i-1} \end{array}\right]}_{World}+∆t\cdot {R\left(\theta_{i-1}\right)}^{-1}\cdot {\left[\begin{array}{c} v_{i-1}\cos\left(\alpha_{i-1}\right)\\ v_{i-1}\sin\left(\alpha_{i-1}\right)\\ \omega_{i-1} \end{array}\right]}_{Robot},$$

where $\left(v_{i-1},\alpha_{i-1},\omega_{i-1}\right)$ is the motion of the mobile robot in the sample i−1. The time lapse ∆t between the samples i−1 and i is the sampling time of its proportional-integral-derivative (PID) motor controllers [112]. ${R\left(\theta \right)}^{-1}$ is the rotation matrix that transforms the robot velocity expressed in the robot frame to the velocity in the world frame:

(2) $${R\left(\theta_{i}\right)}^{-1}=\left[\begin{array}{ccc} cos(\theta_{i})\; & -sin(\theta_{i}) & 0\\ sin(\theta_{i}) & cos(\theta_{i}) & 0\\ 0 & 0 & 1 \end{array}\right].$$

In the case of the APR-02 mobile robot, the instantaneous motion $M_{i}=(v_{i},\alpha_{i},\omega_{i})$ required to follow a planned path is computed using the procedure described in [113].

### 2.2. Reference Map

**Figure 2:**
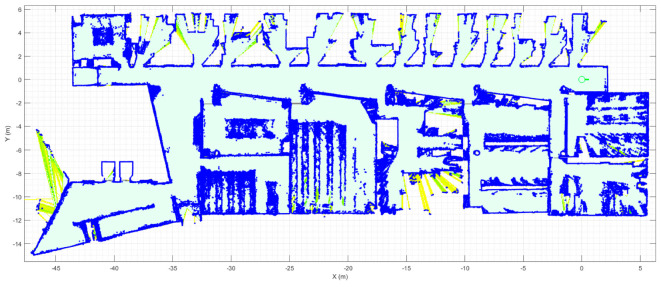
Reference map of one floor of the Polytechnic School at the University of Lleida (Spain). The blue dots are the 2D point cloud map of the floor and the light green surface details the navigable area. Other surface colors depict likely navigable areas.

Figure 2 shows the reference map used in this paper. The map shows the 2D layout of one floor of the multi-story building at the Polytechnic School of the University of Lleida (Spain). The map contains a 2D point cloud (blue points) obtained with 2D LiDAR and a background grid map detailing the navigable areas (light green area). The building has five floors and two elevators. The layouts of floors one, two (Figure 2) and three are very similar, whereas the layouts of the ground floor and the basement have some differences.

This paper only uses the reference map to illustrate the application of path-planning and motion-planning algorithms, so the simplified assumption is that all the plants of the building have the same layout (as shown in Figure 2). The point cloud may seem noisy, but this is how a mobile robot using 2D LiDAR perceives an unstructured environment. The use of a point cloud as a map by a mobile robot using 2D LiDAR provides the mobile robot’s precise self-localization and navigation through narrow areas such as doors. For more specific details on how to obtain the map of the floor, please refer to [114]. Finally, this work is focused on addressing the problem of path planning in a multi-story building; the practical assessment of the APR-02 mobile robot as a delivery robot will be addressed in future works.

### 2.3. Navigation Tree

The navigation tree proposed in this work for multi-story path planning is a manually created graph combining nodes (pick-up and drop-off points) and segments (weighted directional distances). The nodes are used to define the physical location of reference placements and other intermediate trajectory positions. Table 1 summarizes the five types of nodes that can be defined in the navigation tree. By default, the nodes are labelled with a letter (S, T, U, E or D) and a sequence number (X). Start (SX) label indicates a node that defines the localization of a main package pick-up point and battery charging station. Trajectory (TX) is a custom auxiliary node used to define intermediate positions within a trajectory. Unique (UX) is a node used to define precise trajectories in narrow areas. Elevator (EX) is a node that precisely defines the localization of a reference point inside an elevator [31]. Destination (DX) is a node that defines the localization of a known destination point for the mobile delivery robot (a drop-off point).

Each node is defined by the following parameters: a unique identification number (ID) with a value between 1 and the total number of nodes, the category of the node (Table 1—node type), the node label (Table 1—graphic representation) and the position (x/y coordinates and floor number) of the node on the map.

The assumption of this work is that a mobile robot can follow a straight trajectory between two nodes linked or connected with a segment. Therefore, when a direct straight connection between two nodes is not possible, the designer must create additional intermediate trajectory nodes on the map to allow for a path based on a concatenation of straight trajectories.

The segments define a straight and collision-free trajectory between two nodes, and the weight is applied to the Euclidean distance between the two nodes. The navigation tree uses directed segments to allow route prioritization depending on the direction of the mobile robot. Table 2 summarizes the four types of segments that can be defined in the navigation tree. Unconnected segments have no link between nodes. Undirected segments are direct paths between two nodes and have the same weight in both trajectory directions. Directed segments define different weights depending on the direction of the trajectory. Blocked segments define an existing undirected or directed segment that has been temporarily blocked because of the detection of an obstacle. The weights defined are multiplied by the Euclidean distance between the nodes, so a greater weight means a longer virtual distance between the nodes. The weights are used to encourage the mobile robot to follow different routes depending on whether the trip is outgoing or returning.

**Figure 3:**
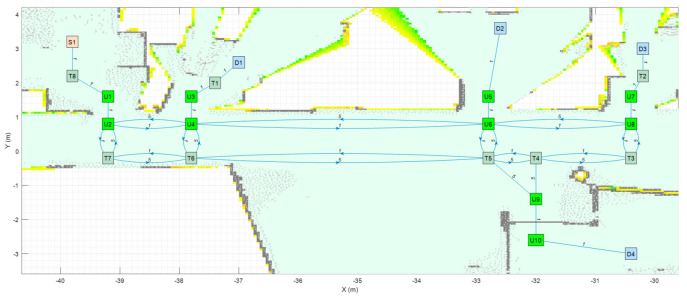
Representation of a small navigation tree defined in one part of the building: S1 is a battery charging station and the main pick-up point; D1…D4 are drop-off points; the pairs U1–U2, U3–U4, U5–U6, U7–U8 and U9–U10 are trajectories required to pass through doors; and T1…T8 are intermediate trajectory positions. The point cloud map is depicted in gray.

As an application example, Figure 3 shows a small navigation tree defined in one part of the building. In this example, S1 is a battery charging station and also the pick-up point. D1…D4 are drop-off destination points usually located inside offices and laboratories. The pairs U1–U2, U3–U4, U5–U6, U7–U8 and U9–U10 are trajectories required to allow the robot to precisely pass through the doors of the destination points. T1…T8 are the intermediate trajectory positions manually created to allow for successful path planning in the building. For instance, in Figure 3, a straight trajectory between the delivery point D3 and the door trajectory point U7 is not possible, so an auxiliary trajectory point T2 has been defined to allow a straight trajectory between U7 and T2 and between T2 and D3 (and vice versa).

The final graph is computed from the weights and trajectories defined in the navigation tree, the Euclidean distance between nodes and the blocked status of the segments described in the matrices N, D and B, respectively. In these matrices, the number of each row coincides with the identification number (ID) of a node. Likewise, following the same order, the number of each column coincides with the ID of a node. The value of one position in the matrix defines a feature (weight, distance or blocked status) of the trajectory going from the row node to the column node. For example, $N_{2,7}=1$ shows that the segment of the navigation tree from node 2 to node 7 has a weight of 1. Similarly, $D_{4,8}=3.45$ shows that the Euclidean distance from node 4 to node 8 is 4.45 m. Finally, $B_{i,j}=0$ shows an unblocked segment between nodes i and j, whereas $B_{i,j}=1$ shows that an obstacle is currently blocking the segment linking the nodes.

The Hadamard [115] (point to point) product of the matrices N, D and B (Equation (3) combines the aforementioned features of each trajectory into a single matrix, called graph G, used by Dijkstra’s algorithm to find the shortest path.

(3) $$G=N_{i,j}\cdot D_{i,j}\cdot {\left(1-B\right)}_{i,j}$$

Since the definition of a navigation matrix N is directed, the resulting graph G is also expected to be directed due to the use of different weights depending on the direction of the trajectory between two nodes.

The blocked status is initialized to 0 by default. The blocked status of a segment $B_{i,j}$ is updated while the robot tries to execute a straight trajectory from node i to j based on the information provided by its LiDAR sensor system. The graph G computed from the navigation tree described in Figure 3 is included in Supplementary Materials.

### 2.4. Dijkstra’s Algorithm

Dijkstra’s algorithm [88] provides the shortest path from one node to every other node on directed graphs [116]. The advantage of Dijkstra’s algorithm is that it provides minimally spanning trees as it explores all possible alternative paths and thus eliminates the uncertainness of heuristic algorithms [117,118]. Dijkstra’s algorithm is commonly used to estimate the shortest trajectory in many mobile robot applications [119,120,121].

Based on a predefined graph G made up of M nodes (also referred as vertices) and E edges (also referred as segments), Dijkstra’s algorithm [88] provides a sub-graph Q∈G starting at an initial node (inode) and ending at a final node (fnode) such that the distance between them ($d_{inode,fnode}$), obtained as the sum of edges defined in the graph G, is minimal:

(4) $$d_{inode,fnode}=\sum_{i=inode}^{fnode}G\left(i,i+1\right),\;\;i\in Q.$$

Q is assumed to be non-empty and includes n vertices, $1\leq n\leq \left|M\right|$, and vertices i and i+1 are adjacent or linked.

**Figure 4:**
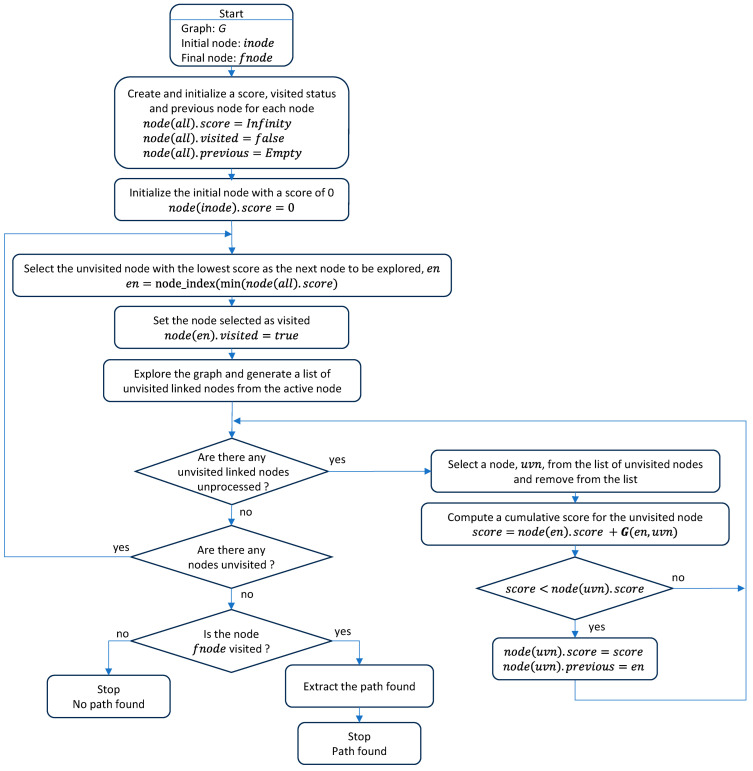
Flowchart of Dijkstra’s algorithm [88].

Figure 4 shows the flowchart of the well-known Dijkstra’s algorithm, which is didactically described in many free video sharing websites such as [122]. In this work, the application of Dijkstra’s algorithm for path planning requires a graph G, the definition of an initial or starting node, inode, and a final or ending node, fnode. 

Dijkstra’s algorithm uses the following parameters of each node: score, visited status and previous node. At the end of the algorithm, score is the cumulated distance travelled to reach the node from the starting node, visited status indicates if the node has been explored and previous node is the preceding linked node at the shortest distance from the current node. The characteristic point of Dijkstra’s algorithm is the determination of the backwards shortest path, starting from the final node to the initial node through the path defined by the previous node values. However, this path is actually travelled starting from the initial node to the final node.

Dijkstra’s algorithm starts by initializing all score values as infinite, the visited status as false and the previous node as empty. Then, the score of the initial node is set to zero. The main loop of the algorithm selects the unvisited node with the lowest score as the next node to be explored (en); in the first iteration, this node is the starting node (that was set to zero for this purpose). The node selected is then marked as visited. The graph is explored to generate a list of unvisited adjacent or linked nodes.

If there are unvisited adjacent nodes, the secondary loop is focused on the exploration of this list of nodes. First, a node is selected as the active node (uvn) and removed from the list. Then, the temporal score of the node uvn is computed, cumulating the score value of en node and the value of the segment from the en node to the node uvn. If this temporal score is lower than the initial score of the node uvn, then its score value is updated with the value of the temporal score and the previous node of en is set as uvn. This process is repeated until the list of unvisited nodes is empty.

The continuation of the main loop step requires the exploration of the visited status of the nodes. If there are unvisited nodes, the loop returns to the step of selecting the unvisited node with the lowest score as the next node to be explored (en) and continues. If there are no unvisited nodes in the graph, the main loop is terminated.

Finally, if the score of the final node is infinite, then no path between the initial and final node has been found. Alternatively, if the final node has been visited with a score that is not infinite, the shortest path between the initial and final node is obtained, generating a sequence that starts at the final node, goes to its previous node and continues exploring the previous nodes until arriving at the initial node. The flowchart of Dijkstra’s algorithm described in Figure 4 is also available as a plain source code in [123].

Table 3 shows the application of Dijkstra’s algorithm to the graph G (Equation (3)), whose navigation tree is shown in Figure 3. The task assigned to the mobile robot is the transportation of a package from the node S1 (the main package pick-up point) to the node D3 (a destination point). The node at which the mobile robot is located is highlighted with a red frame. UX nodes are used to depict precise trajectories that must be followed to navigate through narrow spaces such as doors, while TX nodes are used to depict auxiliary trajectory nodes. The first row of Table 3 simulates the start of a package delivery task with the mobile robot being placed at the node S1. The application of Dijkstra’s algorithm from S1 to D3 provides the path S1–T8–U1–U2–U4–U6–U8–U7–T2–D3, and the mobile robot starts to follow this path checking if the trajectory to the next node is blocked. The second row of Table 3 shows a case in which the mobile robot has reached the node U6 and then detects that the trajectory from node U6 to node U8 is blocked by an obstacle. In this case, the application of Dijkstra’s algorithm from U6 to D3 provides a new path, U6–T5–T4–T3–U8–U7–T2–D3, and the mobile robot starts to follow this path, checking if the trajectory to the next node is blocked. In the third row of Table 3, the mobile robot has arrived at the destination node D3 to deliver the package. The fourth row of Table 3 assumes that the package has been delivered and that the mobile robot must return to the main package pick-up point S1. In this case, the application of Dijkstra’s algorithm from D3 to S1 provides the path D3–T2–U7–U8–T3–T4–T5–T6–T7–U2–U1–T8–S1, and the mobile robot starts to follow this path, checking if the trajectory to the next node is blocked. Finally, the fifth row of Table 3 represents the ideal case in which the mobile robot has completed the return journey without encountering any obstacles blocking any segments. In summary, the detection of an obstacle blocking a segment of the planned path requires the classification of this segment as blocked, the recalculation of the graph (Equation (3)) and the recalculation of the shortest path to the destination using Dijkstra’s algorithm. In future works, the artificial potential field (APF) [124] algorithm will be applied to decide if an obstacle blocking the planned path can be safely avoided [125] using simple maneuvers instead of calculating an alternative path.

## 3. Implementation

This section describes the implementation of the path-planning procedure proposed in this paper, which is based on a predefined navigation tree of the multi-story building.

### 3.1. Predefined Navigation Tree

**Figure 5:**
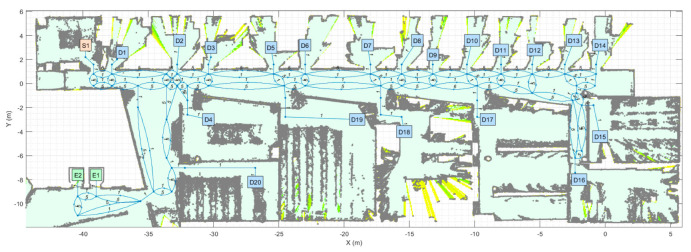
Navigation tree defined in one representative floor of the building of the Polytechnic School of the University of Lleida (Spain). The point cloud map is depicted in gray. S1 is the main package pick-up point of the floor. D1…D20 are the possible destination points located inside offices and laboratories.

The map of one representative floor of the building (Figure 2) is used as a reference to manually create a feasible navigation tree. Figure 5 shows the navigation tree created to develop the path planning of a mobile delivery robot in the multi-story building. As stated in Section 2.2, this work assumes that all the floors of the building have the same layout to simplify our interpretation of the navigation results. The assumptions made to create the navigation tree are as follows:

- The nodes of the navigation tree depict the position of the main pick-up points, destination points, doors and elevators. The nodes are precisely referenced in the point cloud map of the floor of the building (Figure 2).
- The segments of the navigation tree depict straight trajectories between the linked nodes. During the creation of the navigation tree, trajectory nodes can be added to define a sequence of straight segments and avoid fixed furniture obstacles.

The navigation tree displayed in Figure 5 has 171 segments and 101 nodes: 1 S node (the main package pick-up point), 46 U nodes (used to define precise trajectories), 2 E nodes (elevators) and 32 T nodes (used to define intermediate trajectories). In Figure 5, the labels of the U and T nodes are not displayed to avoid covering segments of the graph. The graph deduced from the navigation tree described in Figure 5 is included in the Supplementary Material. An indicator of the complexity of the graph is its density (δ), which is obtained from its number of segments $\left|E\right|$ and its number of nodes $\left|M\right|$:

(5) $$\delta =\frac{\left|E\right|}{\left|M\right|\cdot (\left|M\right|-1)}=\frac{171}{101\cdot (101-1)}=0.0169$$

When the density is within the range of 0≤δ≤1/2, the graph is considered sparse. In this case, the density obtained is in the lowest part of this range, so the graph is sparse and does not represent a computational time challenge for Dijkstra’s algorithm to find the shortest path from one node to any other node.

The memory requirements to store the matrices N, D and B and the graph G of each floor of the building (Equation (3)) are as follows:

(6) $$Memory\_one\_floor=4\cdot \left(\left|M\right|\cdot \left|M\right|\right)=4\cdot \left(\left|M\right|\cdot \left|M\right|\right)=40,804\;FP64,$$

where FP64 is the double-precision floating point format value defined by IEEE 754-2019 [126]. The building with five floors requires the replication of this memory structure in each floor, using a total of 204,020 FP64.

The memory required to compute Dijkstra’s algorithm is determined by the number of nodes in the graph and the parameters defined in each node. In this multi-story application, Dijkstra’s algorithm is called once per floor, computing the shortest path in each floor separately. The algorithm is not called to compute the path between floors, because navigation between floors is only possible through the elevator. Therefore, Dijkstra’s algorithm is only applied to find the shortest path in each floor of the building. For example, a package delivery task starting at the package pick-up node S1 located on the ground floor (F0) and with a destination at D16 on the third floor (F3) defines two paths: the first one from the pick-up point S1–F0 to the elevator E1–F0 and then from the elevator E1–F3 to the destination D16–F3, and vice versa to return. Hence, Dijkstra’s algorithm is only used twice: from S1–F0 to E1–F0 and from E1–F3 to D16–F3, and not between elevator positions from E1–F0 to E1–F3. The algorithm is used twice more to return.

In summary, the memory required by Dijkstra’s algorithm is computed from the number of nodes ($\left|M\right|$), the parameters P associated with each node (the score, previous node and visited status) and the list of nodes linked to the current node explored during the iterative search:

(7) $$Memory\_Dijkstra<\left(\left|P\right|+1\right)\cdot \left|M\right|=\left(3+1\right)\cdot \left|101\right|=404\;FP64$$

The advantages of manually defining the navigation tree on each floor are the reduction in the graph size and the low computational resources required to find the best path, which can be updated when any obstacle blocks the current path. These advantages make this proposal ideal for its implementation in indoor autonomous mobile delivery robots using central processing units based on microcontrollers or low-performance computers.

**Figure 6:**
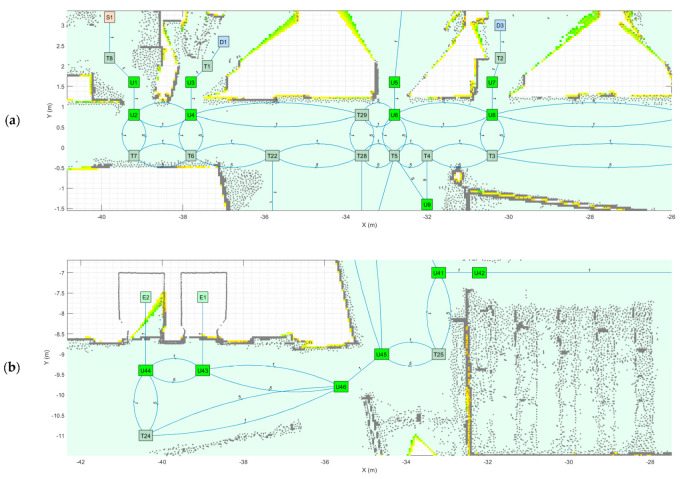

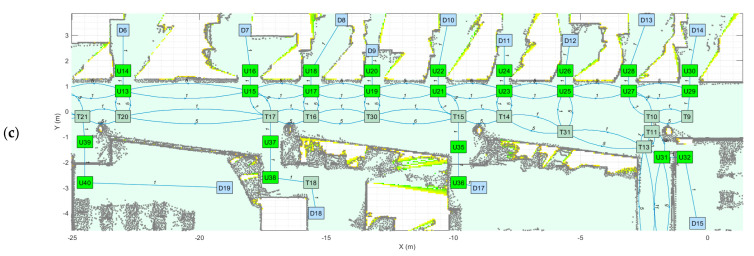
Details of different parts of the navigation tree showing the auxiliary trajectory nodes: (**a**) main package pick-up point S1, hall and initial part of the corridor; (**b**) elevator zone; and (**c**) end of the corridor.

Figure 6 details the navigation tree displayed in Figure 5. The directed graph is proposed to incentivize the robot to move close to the walls following different trajectories when going and returning from the destination. Figure 6a details the navigation tree connecting the hall and corridor. Note that although the lines between the nodes are curved (to better show the directed weights of the navigation tree), all trajectories between the nodes are straight. The concentration of points in the left part of the hall map correspond to a decorative flowerpot. The node T22 shown in Figure 6a is defined specifically to avoid this flowerpot. The node with more segments is T5 (Figure 6a), which is linked to the nodes T28, U6, T4, U9 and T27 (T27 is not visible in Figure 6a). Figure 6b details the navigation tree around the elevators. The trajectories to enter and exit the elevators are defined by U43-E1-U43 and U44-E2-U44 [31], and the mobile delivery robot must be able to wirelessly interact with them [30]. Finally, Figure 6c details the navigation tree at the end of the corridor, which incentivizes the robot to move following different trajectories when going and returning from its destination to simplify and be compatible with the circulation of people and other robots in the corridor.

### 3.2. Path Planning in a Multi-Story Building

In mobile robotics, path planning is a procedure that computes a basic collision-free path from a starting point to a destination point using known static information [127,128]. This work assumes that a mobile delivery robot has access to a manually created navigation tree where the nodes detail the position of all expected pick-up and drop-off points, while the segments describe collision-free straight trajectories between the linked nodes. The assignment of a task to a mobile delivery robot consists of defining a destination node to transport and drop off a package from a known pick-up point. Once one task is assigned, the mobile robot must analyze if this task requires navigating between floors and adding the elevator as an intermediate node to be visited. Then, Dijkstra’s algorithm provides a collision-free path for the mobile robot. The mobile robot uses the information of its onboard LiDAR and the point cloud map for self-localization in order to follow the planned path. Finally, in cases in which it detects an obstacle in one segment of the path during transportation, the mobile robot must update the segment as blocked and use Dijkstra’s algorithm again to obtain another collision-free path.

As an example, Table 4 shows the definition of go and return tasks for the mobile delivery robot. Task 1 is a trajectory in which the mobile robot is at the node S1 of the ground floor (labeled as Floor0 and F0) and has to transport a package up to the node D16 of the first floor (labeled as Floor1 and F1). Task 2 is a return trajectory in which the mobile robot is at the node D16 of the first floor and returns to the node S1 of the ground floor. In both tasks, the initial and destination nodes are at different floors, so the mobile robot must take an elevator to complete the transportation task. In consequence, one elevator node has been added in the sequence of nodes to visit by the mobile robot. In Table 4, elevator 1 (E1 node) is prioritized to go up and elevator 2 (E2 node) is prioritized to go down. The elevator nodes are shared between floors.

**Figure 7:**
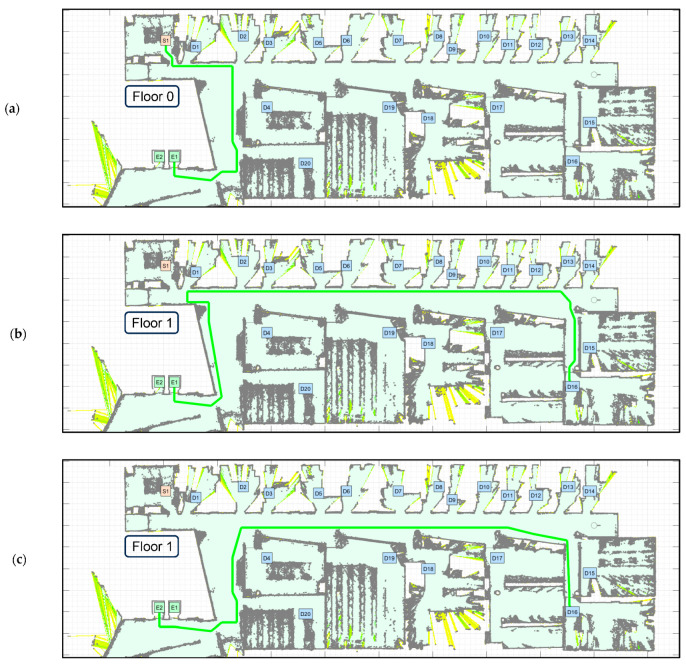

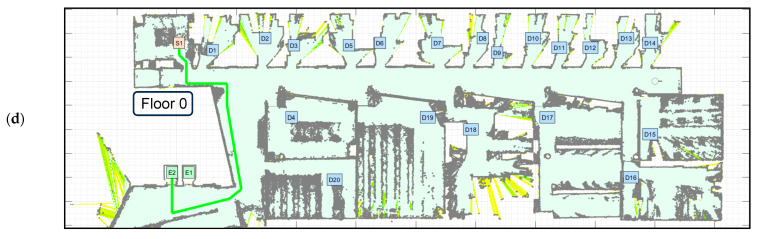
Path planning of the tasks defined in Table 4. Task 1: (**a**) go from the main pick-up point S1–F0 (on the ground floor) to E1–F0 and take the elevator; (**b**) go from E1–F1 (on the first floor) to D16–F1. Task 2: (**c**) return from D16–F1 to E2–F1 and take the elevator; and (**d**) go from F2–F0 (on the ground floor) to S1–F0 (main pick-up point).

Figure 7 shows the path proposed by Dijkstra’s algorithm to complete the tasks defined in Table 4. Task 1 comprises two sub-paths: starting from S1–F0 (on the ground floor) to E1–F0 and taking the elevator (Figure 7a), and exiting the elevator on the first floor and going from E1–F1 to D16–F1 (Figure 7b). Task 2 also comprises two sub-paths: starting from D16–F1 (on the first floor) to E2–F1 and taking the elevator (Figure 7c), and exiting the elevator on the ground floor and going from E2–F0 to S1–F0 (Figure 7d). In both cases, the assumption is that the mobile robot can wirelessly communicate with the elevator in order to call it and select a destination floor [31].

### 3.3. Motion Planning in a Multi-Story Building

In mobile robotics, motion planning is a procedure that assumes that the motion of a mobile robot must face dynamic obstacles not considered during path planning. These obstacles can be dynamic, such as people, companion animals and other mobile robots, or stationary, such as furniture added after the creation of the map and navigation tree. 

**Figure 8:**
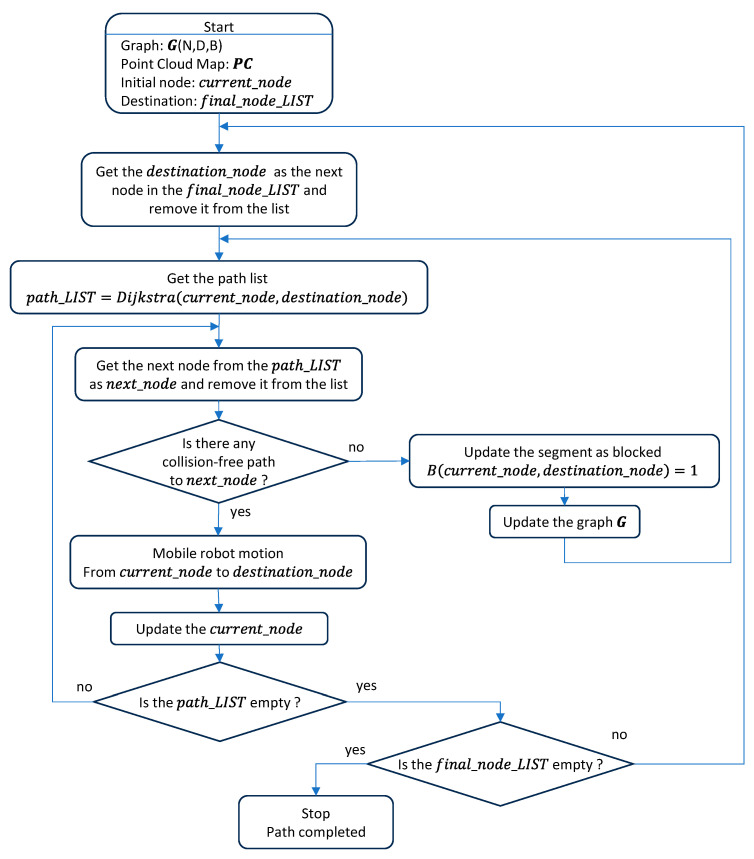
Flowchart of the motion-planning algorithm.

Figure 8 shows the flowchart of the motion-planning procedure proposed for the mobile delivery robot. This procedure requires the graph G, the point cloud PC, the node in which the mobile robot is located (current_node) and a list of the nodes to be visited (final_node_LIST).

The main loop of the algorithm starts initiating the intermediate destination node (destination_node) with the next node listed in final_node_LIST and removing it from the list. At the end of the main loop, the score is the cumulated distance from the starting node to the destination node. The secondary loop of the algorithm starts by getting the shortest path from the current node to the intermediate destination node to be visited using Dijkstra’s algorithm and stores the result in a new list with the intermediate nodes (path_LIST). The tertiary loop extracts the next node (next_node) to be visited by the mobile robot from the path_LIST. After this step, the sensors of the mobile robot must validate if there is a collision-free path to next_node. If any obstacle is detected in this path, the segment must be classified as blocked and the path_LIST must be updated. If no obstacle is detected, then the mobile robot must follow the trajectory defined from its current position (current_node) to the position defined by next_node and update the value of the current_node when finishing the displacement.

After completing this loop, the path_LIST must be checked to select the next partial destination. If the path_LIST is empty, then the final_node_LIST must be checked to get a new destination_node. The planned path is completed when the final_node_LIST is empty.

## 4. Results

The main simulation results presented in this section correspond to the tasks presented in Table 4. The path followed by the robot (shown in Figure 7) has been obtained with Dijkstra’s algorithm [88] (Figure 4) and the graph created from the navigation tree (Figure 5 and Equation (3)). The path-tracking performances of the simulated mobile robot emulate the omnidirectional motion capabilities of the reference APR-02 mobile robot (Equation (1) and Figure 1) [79]. The motion required to follow a planned path is computed using the procedure described in [113].

**Figure 9:**
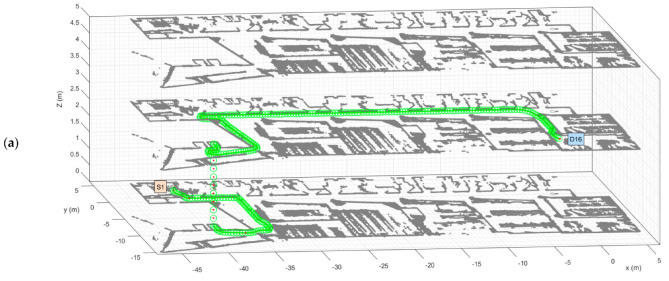

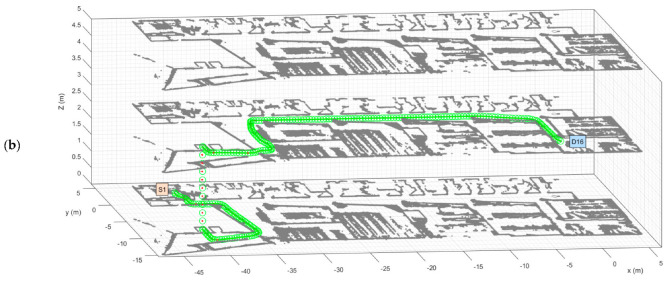
Simulated routing of the mobile delivery robot: (**a**) developing task 1 defined in Table 4: starting at S1–F0 (ground floor) and going to D16–F1 (first floor); (**b**) developing task 2 defined in Table 4: starting at D16–F1 (first floor) and going to S1–F0 (ground floor). Only three of the five floors of the building are shown. The red dots and green circles represent the position of the robot.

Figure 9 shows a 3D routing simulation of the operation of the mobile delivery robot in the studied multi-story building. The number of graphical features displayed in the figures has been minimized to better show the planned path and the simulated motion. Figure 9a shows the mobile delivery robot developing task 1 as defined in Table 4: starting at the main package pick-up point S1–F0 (on the ground floor) and going to the destination point D16–F1 (on the first floor). Figure 9b shows the mobile delivery robot developing task 2 as defined in Table 4: starting at the destination point D16–F1 (on the first floor) and returning to the main package pick-up point S1–F0 (on the ground floor). In both figures, the movement of the mobile robot inside the elevator has also been simulated.

**Figure 10:**
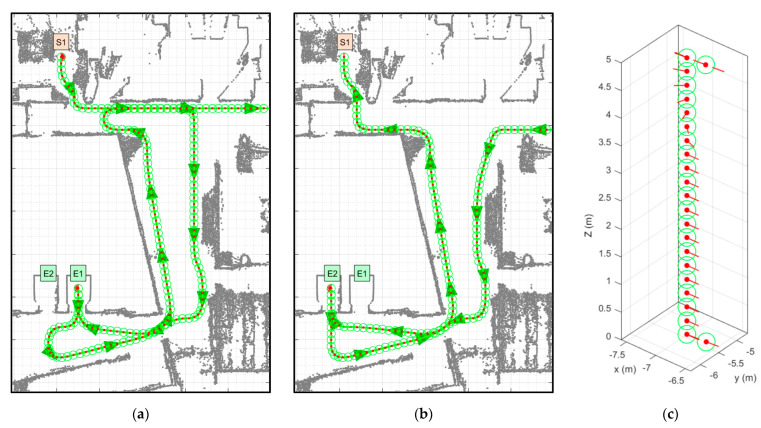
Details of the motions simulated: (**a**) top view of the motion of the mobile robot starting at node S1 on the ground floor, entering the elevator E1, exiting on the first floor and going to node D16; (**b**) top view of the motion of the mobile robot coming from D16 on the first floor, entering the elevator E2, exiting it on the ground floor and going to node S1; (**c**) 3D view of the motion of the mobile robot inside one elevator, entering on the second floor and exiting on the ground floor. The red dots, red lines and green circles represent the position and orientation of the robot.

Figure 10 shows top view details of the motions simulated. Figure 10a shows the motion of the mobile delivery robot initiating the task at the node S1–F0 on the ground floor, exiting from the main package pick-up point, following the path close to the robot’s left wall, crossing the corridor, going through the hall until it reaches the elevator lobby and going to the elevator E1–F0. Before arriving at this point, the mobile robot must call the elevator E1 to avoid having to wait. The mobile robot enters the elevator facing inwards, sets a destination floor, and turns 180° during its journey to prepare to exit (see Figure 10c). The exiting trajectory is different from the entrance trajectory as a way to reduce trajectory interferences with other mobile robots. Once out of the elevator, the mobile robot goes to the hall and follows the path close to its left wall until arriving at the corridor and follows a path close to its left wall up to the destination point D16–F1.

Figure 10b shows the motion of the mobile delivery robot returning from the node D16–F1 on the first floor, following a path close to its left wall until arriving at the hall, following a path close to its left wall until reaching the elevator lobby and going to the elevator E2–F1. Again, before arriving at this point, the mobile robot must call the elevator E2 to avoid having to wait. In this simulated example, elevator E1 was prioritized to go up and E2 was prioritized to go down, but this selection can be random or based on elevator proximity. Again, the mobile robot enters the elevator facing inwards, so it must turn 180° during its journey to prepare to exit (see Figure 10c). Once out of the elevator, the mobile robot goes through the lobby, enters the hall and follows the path close to its left wall until arriving at the main package pick-up point of the ground floor S1–F0. Specifically, Figure 10c details the motion of the mobile robot inside the elevator: entering on the second floor and exiting on the ground floor. Once inside the elevator, the mobile robot has to rotate 180° to face the elevator door, detect its opening and exiting through the door. This rotation is strictly necessary due to the fact that the APR-02 mobile robot uses a 2D LiDAR that does not measure the rear area of the robot, so it has to rotate to be able to detect the opening of the elevator door and avoid colliding with any person entering the elevator.

Table 5 summarizes the routing results obtained in 40 complementary simulations. Table 5 shows an estimation of the length of the trajectory that the robot has to travel to complete the delivery of a package starting at the main pick-up point of the building (node S1–F0), going to a delivery point (node DX, where X is the node number from 1 to 20) and returning to the main pick-up point (S1–F0). Twenty tasks have been evaluated. Table 5 shows the cumulated length of each trajectory in case the destination node is on the same floor as the initial node, the cumulated length in case the destination node is on another floor of the building and the difference between both of these cases due to the navigation between floors. As illustrated in Figure 7, each transportation and delivery task listed in Table 5 requires two or four sub-paths depending on the floor of the destination. If the initial node and the destination node are on the same floor, the sub-paths go from S1–F0 to DX–F0 (the outgoing path) and from DX–F0 to S1–F0 (the return path). If the initial node and the destination node are on different floors, the sub-paths go from S1–F0 to E1–F0 (the path to take the elevator configured for upwards travel), from E1–FY to DX–FY (the path from the elevator to the destination at any floor Y), from DX–FY to E2–FY (the return path to take the elevator configured for downwards travel) and from E2–F0 to S1–F0 (the return path). 

The cumulated length shown in Table 5 has been obtained using Dijkstra’s algorithm to obtain the shortest path to complete each sub-path. The navigation between floors requires the mobile robot to travel a few meters further. For example, when the destination is close to the elevator’s zone (task T20 of Table 5), the robot travels 41.87 m further. When the destination is very close to the starting point (task T1 of Table 5), the robot travels 89.86 m further. Nevertheless, the additional distance to be travelled is 78.77 m for the majority of the tasks (Table 5). This difference does not depend on the destination floor because the mobile robot is static and only rotates while it is in the elevator. 

Complementarily to Table 5, Table 6 shows the distance–task matrix (DT matrix) of the multi-story building which can be used to generally estimate the energy consumption of the mobile delivery robot [100]. The distance–task matrix describes the path length of all possible sub-paths, starting at any node up to any node in the same floor. In this work, the assumption is that all floors have the same layout, so one distance–task matrix is representative of all the plants of the building. Nevertheless, in a general case, each floor of the building requires its specific DT matrix. 

Table 6 was created using Dijkstra’s algorithm row by row. For example, in the first row, the starting node is S1 and Dijkstra’s algorithm provides the shortest path from S1 to all nodes connected in the graph to guarantee finding the shortest path. This means that Dijkstra’s algorithm is used only 23 times to compute all of Table 6. Additionally, it is expected that the distance–task matrix does not change as the building does not change. Hence, the DT matrix can be used as a look-up table to quickly plan complex sequences of tasks without ever having to use Dijkstra’s algorithm.

In Table 6, the task of starting and ending at the same node without changing the floor has been highlighted in yellow for reference. Once available, the DT matrix can be used to obtain the first estimation of the distance required to complete any single task or multiple tasks defined using a node list (nodeList):

(8) $$TotalEnergy=\sum_{i=1}^{p-1}DT\left(nodeList(i),nodeList(i+1)\right)\cdot EPM+Elevator\_time\cdot EPT,$$

where p is the number of nodes in the list, EPM is the average energy expended by the robot in each linear meter of travel, Elevator_time is the average travel time spent in the elevator and EPT is the energy expended by the robot in each unit of time when it is not moving.

As an example, the length of the trajectory required to implement task T3 of Table 5 is defined by the nodeList=[S1,D3,S1] and is obtained using the following:

(9) $$d_{T3}=DT\left(S1,D3\right)+DT\left(D3,S1\right)=13.7\;m+15.7\;m=29.4\;m.$$

Alternatively, the cumulated trajectory length for the same task T3 of Table 5 requiring navigation between floors, for example to node D3 on floor 1, is defined by the nodeList=[S1-F0, E1-F0→F1, D3-F1, E2-F1→F0,S1-F0]. In this case, it is considered that elevator E1 is defined to go upwards and elevator E2 to go downwards. The cumulated path length is then obtained using the following:

(10) $$d_{T3-F1}=DT\left(S1,E1\right)+DT\left(E1,D3\right)+DT\left(D3,E2\right)+DT\left(E2,S1\right)=26.0+32.6+23.9+25.6=108.1\;m$$

## 5. Discussion and Conclusions

This paper addresses the path planning of an autonomous mobile robot that has to transport and deliver small packages in a multi-story building. The mobile robot’s path must drive the robot from a known starting point to a destination point. The main assumptions made are that the building is equipped with remotely controlled elevators and that the door of the target destination is open, so the mobile robot does not need to open any door to deliver a package. This proposal is based on the manual definition of a navigation tree combining the spatial and operational information required to move within a multi-story building. The manual definition of this static navigation tree has similarities to painting reference lines on the floor. However, instead of following a line painted on the floor, the mobile delivery robot uses 2D LiDAR and a reference point cloud map for self-localization, precise path tracking and obstacle avoidance. The navigation tree is used to build a graph of each floor of the building and plan the path of the robot using Dijkstra’s algorithm with the elevator nodes as the connectors between floors. Each graph is created by combining the weighted segments defined in the navigation tree (used to prioritize routes), the real distance between the nodes and an additional matrix used to highlight the segments blocked by dynamic objects. The graph is visually represented as a combination of directed and undirected segments to prioritize trajectories depending on the direction of the motion.

The graph is based on the definition of the five types of nodes (see Table 1) labelled with a letter (S, T, U, E or D) and an identification number (X). Start (SX) defines a main charging and package distribution point. Trajectory (TX) defines intermediate points required to specify straight trajectories between two nodes of the graph. Unique (UX) defines nodes required to specify special and precise structural trajectories that cannot be modified. Elevator (EX) defines the placement of an elevator. Destination (DX) defines the expected locations of all possible destinations on each floor of the building. There are four segment types that can be used in the graph (see Table 2). Unconnected segments have no direct path between nodes. Undirected segments have no direction differentiation. Directed segments define a weight depending on the direction of the trajectory. Blocked segments are used to represent an existing directed or undirected segment that is temporarily blocked by an unexpected dynamic obstacle.

The navigation tree used in this paper has directed segments with a weight of 1 to depict a priority direction and a weight of 5 to depict a non-priority direction. A graph is created by multiplying the weights of the navigation tree by the Euclidean distance between nodes. Then, Dijkstra’s algorithm guarantees that the shortest path between nodes is obtained considering the directions prioritized by the weighted segments. The information of the obstacles detected during motion is used to update the blocked matrix (see Table 3) and the graph, which is then used to search for a new shortest path to the destination. The weighted graph defines trajectories with differentiated outgoing and returning trajectories that are expected to be compatible with the typical movement of people and other mobile robots in the building.

As a reference application example, this paper details the navigation tree manually defined to describe the layout of one representative floor of the Polytechnic School at the University of Lleida. This example navigation tree has 101 nodes representing expected pick-up and drop-off points. These nodes are connected with 171 segments that define a sparse graph, which is explored quickly by Dijkstra’s algorithm to return the shortest path between any two nodes. At this point, it should be noted that this sparse graph reduces the size and connections of the graph by two or three orders of magnitude compared with the dense graphs automatically created in a grid map application [85,87,96], which makes it unnecessary to use heuristic searching methods such as the A-star, that does not guarantee finding the best path [91,92] (as it performs a best-first search [93]).

Therefore, the size of the resulting sparse graph allows an autonomous mobile robot to recalculate in real time the shortest path to the destination in case the planned trajectory is temporarily blocked by an obstacle. For the sake of completeness, this paper describes the flowchart of the implementation of Dijkstra’s algorithm (Figure 4) and also provides the implementation of a reference source code [123].

In general, planning the path of a task assigned to a mobile delivery robot operating in a multi-story building requires four sub-paths (Figure 7): a sub-path from the initial pick-up node to the elevator and another sub-path from the elevator to the destination point; and a return sub-path from the destination point to the elevator and from the elevator to the main pick-up point. Motion planning is the dynamic task that is in charge of following the planned path based on the motion and self-localization performances of the mobile robot. Motion planning assumes that the robot is able to detect obstacles blocking its displacement. This paper describes the flowchart of the motion-planning algorithm (Figure 8) that was developed to simulate the trajectories of a mobile delivery robot. This motion planning will be assessed in a future application of the APR-02 mobile robot operating as a delivery robot. 

The simulated routing results have demonstrated the effectiveness of the path-planning procedure applied to an autonomous delivery robot performing in a multi-story building (Figure 9). Additionally, in the multi-story building studied in this paper, our analysis of the cumulative path length of all possible trajectories from a common starting point to all delivery nodes shows that navigation between floors generates an increase in the path length with a median of 78.77 m.

This paper also proposes the computation of a distance–task matrix that can be used to quickly estimate the path length between any two nodes on each floor and using the elevators to navigate between the floors. The advantage provided by the use of the distance–task matrix is that the heavy computations required to create this matrix only need to be performed once. Hence, the distance–task matrix, used as a look-up table, saves time during planning as it avoids the need to repetitively use Dijkstra’s algorithm to estimate the length of each sub-path required to complete a specific task or a sequence of tasks. In this work, all floors of the building have the same layout and use the same distance–task matrix; in a general case with different layouts, each floor will need a specific navigation tree and will have a different distance–task matrix. Additionally, the estimated path length can be used to directly estimate the energy consumption of a mobile delivery robot [100] performing a task or a sequence of tasks.

The final conclusion of this paper is that a predefined navigation tree can be used for mobile robot path planning in a multi-story building. Specifically, the application of Dijkstra’s algorithm to the sparse graph created from the navigation tree and the real Euclidean distances between nodes provides the shortest path between any two nodes of the graph without any outstanding memory or computational requirements. Finally, the possibility of differentiating outgoing and returning trajectories and prioritizing motion directions is expected to be compatible with the typical movement of people and other mobile robots in the building.

As a future work, the path-planning method proposed in this paper will be implemented using the prototype APR-02 mobile robot, which will be reconfigured as a delivery robot to be assessed in a multi-story building. The problem of opening and closing the doors of rooms with a delivery robot will be also addressed in future works.

## Figures and Tables

**Table 1:** Types of nodes used in the navigation tree.

Graphic Representation	Node Type	Description	Position Editable in the Map
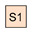	Start	Main package pick-up node and battery charging station	No
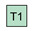	Trajectory	Intermediate trajectory node	Yes
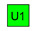	Unique	Special node used to define precise trajectories, for example, to pass through open doors	No
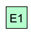	Elevator	Placement of an elevator	No
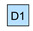	Destination	Destination node	Yes

**Table 2:** Types of segments used in the navigation tree.

Segments		Weight	
Graphic Representation	Type	From S1 to D1	From D1 to S1
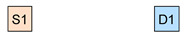	unconnected	Infinite	Infinite
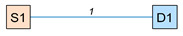	undirected	1.0	1.0
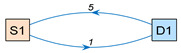	directed	1.0	5.0
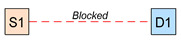	blocked	Infinite	Infinite

**Table 3:** Example of Dijkstra’s algorithm in the path planning of a mobile delivery robot.

Current Node of the Robot	Is Next Segment Blocked?	Current Delivery		Planned Path
		From	To	
S1	No	S1 (Package pick-up point)	D3 (Destination point)	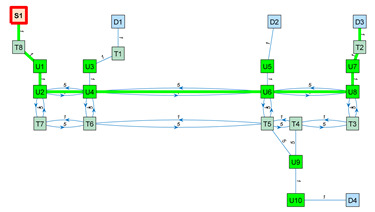
U6	Yes (Compute a new path from U6)	U6	D3	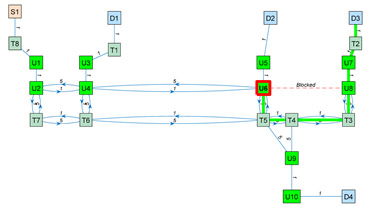
D3 (Package delivery)	-	U6	D3	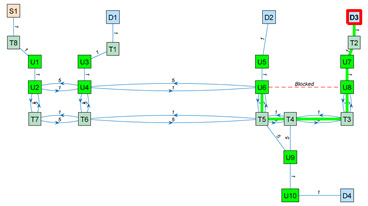
D3 (Return)	No	D3 (Return to starting point)	S1	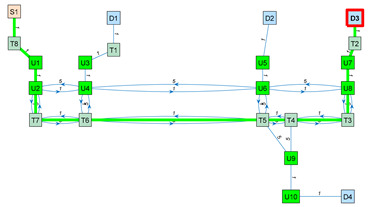
S1 (Arrival at the departure point)	-	D3	S1	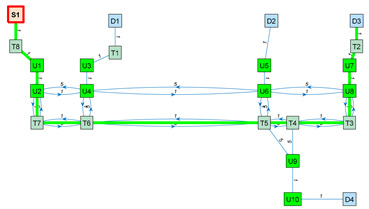

**Table 4:** Task definition example for the mobile delivery robot.

Task	Initial Node	Destination Node	Sequence of Nodes to Visit
1 (go)	S1-F0	D16-F1	1: S1-F0 2: E1-F0→F1 3: D16-F1
2 (return)	D16-F1	S1-F0	1: D16-F1 2: E2-F1→F0 3: S1-F0

**Table 5:** Path length of the available tasks for the mobile delivery robot in the multi-story building.

Task	Initial Node	Destination Node	Final Node	Cumulated Path Length When Origin and Destination Are		
				On the Same Floor	On Different Floors	Difference
T1	S1	D1	S1	12.27 m	102.13 m	89.86 m
T2	S1	D2	S1	25.72 m	104.49 m	78.77 m
T3	S1	D3	S1	29.36 m	108.13 m	68.77 m
T4	S1	D4	S1	29.24 m	108.01 m	78.77 m
T5	S1	D5	S1	40.41 m	119.18 m	78.77 m
T6	S1	D6	S1	44.50 m	123.27 m	78.77 m
T6	S1	D7	S1	55.60 m	134.37 m	78.77 m
T8	S1	D8	S1	60.76 m	139.53 m	78.77 m
T9	S1	D9	S1	62.50 m	141.27 m	78.77 m
T10	S1	D10	S1	71.26 m	150.03 m	78.77 m
T11	S1	D11	S1	73.70 m	152.47 m	78.77 m
T12	S1	D12	S1	79.20 m	157.97 m	78.77 m
T13	S1	D13	S1	87.68 m	166.45 m	78.77 m
T14	S1	D14	S1	90.16 m	168.93 m	78.77 m
T15	S1	D15	S1	91.09 m	169.86 m	78.77 m
T16	S1	D16	S1	95.38 m	174.15 m	78.77 m
T17	S1	D17	S1	72.43 m	151.20 m	78.77 m
T18	S1	D18	S1	61.24 m	140.01 m	78.77 m
T19	S1	D19	S1	52.19 m	130.96 m	78.77 m
T20	S1	D20	S1	50.69 m	92.56 m	41.87 m

**Table 6:** Distance–task matrix of the target multi-story building showing the distances (in meters) obtained with Dijkstra’s algorithm from one initial or starting node (row) to a destination node (column). The matrix is not symmetric due to the use of a weighted and directed navigation tree. The tasks starting and ending at the same node (non-motion tasks) have been highlighted in yellow for reference.

	S1	D1	D2	D3	D4	D5	D6	D7	D8	D9	D10	D11	D12	D13	D14	D15	D16	D17	D18	D19	D20	E1	E2
**S1**	0.0	6.1	11.9	13.7	14.3	18.7	21.2	26.3	29.4	30.2	34.1	35.8	38.3	41.7	43.6	45.3	48.2	36.0	30.4	25.8	23.7	26.0	27.4
**D1**	6.1	0.0	9.9	11.7	12.4	16.7	19.3	24.3	27.4	28.3	32.1	33.9	36.3	39.7	41.6	43.4	46.3	34.0	28.4	23.9	21.8	24.1	25.5
**D2**	13.9	11.9	0.0	7.4	8.1	12.4	15.0	20.0	23.1	24.0	27.8	29.6	32.0	35.4	37.3	39.1	42.0	29.7	24.1	19.6	18.4	20.7	22.1
**D3**	15.7	13.7	9.4	0.0	8.9	9.4	12.0	17.0	20.2	21.0	24.9	26.6	29.0	32.5	34.4	36.1	39.0	26.7	21.1	16.6	20.2	22.5	23.9
**D4**	14.9	12.9	8.7	10.5	0.0	15.5	18.0	23.1	26.2	27.0	30.9	32.6	35.1	38.5	40.4	42.1	45.0	32.8	27.2	22.6	19.5	21.8	23.2
**D5**	21.7	19.8	15.5	12.5	14.9	0.0	7.0	12.0	15.1	16.0	19.9	21.6	24.0	27.4	29.3	31.1	34.0	21.7	16.1	11.6	26.3	28.6	30.0
**D6**	23.2	21.3	17.0	14.0	16.4	8.5	0.0	9.8	12.9	13.8	17.6	19.4	21.8	25.2	27.1	28.9	31.8	19.5	13.9	13.1	27.8	30.1	31.5
**D7**	29.3	27.4	23.1	20.1	22.5	14.6	12.9	0.0	7.9	8.8	12.7	14.4	16.8	20.2	22.1	23.9	26.8	14.5	8.9	19.2	33.9	36.2	37.6
**D8**	31.4	29.4	25.1	22.2	24.6	16.6	14.9	9.4	0.0	7.1	11.0	12.7	15.1	18.6	20.5	22.2	25.1	12.8	11.0	21.2	35.9	38.2	39.6
**D9**	32.2	30.3	26.0	23.0	25.4	17.5	15.8	10.3	9.1	0.0	7.0	8.8	11.2	14.6	16.5	18.3	21.2	8.9	11.8	22.1	36.8	39.1	40.5
**D10**	37.2	35.2	30.9	28.0	30.4	22.4	20.7	15.2	14.1	10.1	0.0	7.4	9.9	13.3	15.2	16.9	19.8	7.5	16.7	27.0	41.7	44.0	45.4
**D11**	37.8	35.9	31.6	28.6	31.0	23.1	21.4	15.9	14.7	10.8	8.9	0.0	6.4	9.8	11.7	13.5	16.4	8.2	17.4	27.7	42.4	44.7	46.1
**D12**	40.9	39.0	34.7	31.7	34.1	26.2	24.5	19.0	17.8	13.9	12.0	9.1	0.0	7.4	9.3	11.1	14.0	11.3	20.5	30.8	45.5	47.8	49.2
**D13**	46.0	44.1	39.8	36.8	39.2	31.3	29.6	24.1	22.9	19.0	17.1	14.2	7.4	0.0	7.7	9.5	12.4	16.4	25.6	35.9	50.6	52.9	54.3
**D14**	46.6	44.6	40.3	37.4	39.8	31.8	30.1	24.6	23.5	19.5	17.7	14.7	12.9	9.1	0.0	10.1	13.0	17.0	26.2	36.4	51.1	53.4	54.8
**D15**	45.8	43.8	39.5	36.6	39.0	31.0	29.3	23.8	22.7	18.7	16.8	13.9	12.1	9.5	10.1	0.0	10.0	16.2	25.4	35.6	50.3	52.6	54.0
**D16**	47.2	45.2	40.9	37.9	40.4	32.4	30.7	25.2	24.0	20.1	18.2	15.3	13.4	12.2	12.8	12.0	0.0	17.5	26.7	37.0	51.7	54.0	55.4
**D17**	36.5	34.5	30.2	27.3	29.7	21.7	20.0	14.5	13.4	9.4	7.5	9.3	11.7	15.1	17.0	18.8	21.7	0.0	16.1	26.3	41.0	43.3	44.7
**D18**	30.9	28.9	24.6	21.7	24.1	16.1	14.4	8.9	12.0	12.9	16.7	18.5	20.9	24.3	26.2	28.0	30.9	18.6	0.0	20.7	35.4	37.7	39.1
**D19**	26.4	24.4	20.1	17.1	19.6	11.6	14.2	19.2	22.3	23.2	27.0	28.8	31.2	34.6	36.5	38.3	41.2	28.9	23.3	0.0	30.9	33.2	34.6
**D20**	27.0	25.0	30.7	32.5	33.2	37.5	40.1	45.1	48.2	49.1	52.9	54.7	57.1	60.5	62.4	64.2	67.1	54.8	49.2	44.7	0.0	17.5	18.9
**E1**	27.0	25.0	30.7	32.6	33.2	37.5	40.1	45.1	48.3	49.1	53.0	54.7	57.1	60.5	62.5	64.2	67.1	54.8	49.2	44.7	22.0	0.0	5.0
**E2**	25.6	23.6	29.3	31.2	31.8	36.1	38.7	43.7	46.9	47.7	51.6	53.3	55.7	59.1	61.1	62.8	65.7	53.4	47.8	43.3	20.6	5.0	0.0

## Data Availability

The data reported in this paper are provided as Supplementary Materials that can be downloaded at: https://www.mdpi.com/article/10.3390/s23218795/s1.

## Supplementary Materials

The following supporting information can be downloaded at: https://www.mdpi.com/article/10.3390/s23218795/s1. This link is a zip file contains the graphs of the application example, the graphs of one floor of the target building and one video showing the simulation results.

## References

[1] Statista Retail E-Commerce Sales Growth Worldwide 2017–2027. https://www.statista.com/statistics/288487/forecast-of-global-b2c-e-commerce-growth/.

[2] Leyerer M., Sonneberg M., Heumann M., Breitner M. (2019). Decision support for sustainable and resilience-oriented urban parcel delivery. EURO J. Decis. Process..

[3] Chen M., Wu P., Hsu Y. (2019). An effective pricing model for the congestion alleviation of e-commerce logistics. Comput. Ind. Eng..

[4] Cardenas I., Beckers J., Vandelslander T., Verhetsel A., Dewulf W. Spatial characteristics of failed and successful Ecommerce deliveries in Belgian cities. Proceedings of the ILS 2016—6th International Conference on Information Systems, Logistics and Supply Chain.

[5] Langevin A., Mbaraga P., Campbell J. (1996). Continuous approximation models in freight distribution: An overview. Transp. Res. Part B Methodol..

[6] Boysen N., Fedtke S., Schwerdfeger S. (2021). Last-mile delivery concepts: A survey from an operational research perspective. OR Spectr..

[7] Jiang L., Mahmassani H. (2014). City logistics. Transp. Res. Rec. J. Transp. Res. Board.

[8] Dablanc L., Diziain D., Levifve H. (2011). Urban freight consultations in the Paris region. Eur. Transp. Res. Rev..

[9] Anderluh A., Hemmelmayr V.C., Rüdiger D. (2020). Analytic hierarchy process for city hub location selection—The Viennese case. Transp. Res. Procedia.

[10] Li L., He X., Keoleian G.A., Kim H.C., De Kleine R., Wallington T.J., Kemp N.J. (2021). Life cycle greenhouse gas emissions for last-mile parcel delivery by automated vehicles and robots. Environ. Sci. Technol..

[11] Himstedt B., Meisel F. (2023). Parcel delivery systems for city logistics: A cost-based comparison between different transportation technologies. Logist. Res..

[12] Mohammad W.A.M., Diab Y.N., Elomri A., Triki C. (2023). Innovative solutions in last mile delivery: Concepts, practices, challenges, and future directions. Supply Chain. Forum Int. J..

[13] Kim J., Moon H., Jung H. (2020). Drone-Based Parcel Delivery Using the Rooftops of City Buildings: Model and Solution. Appl. Sci..

[14] Fragapane G., de Koster R., Sgarbossa F., Strandhagen J.-O. (2021). Planning and control of autonomous mobile robots for intralogistics: Literature review and research agenda. Eur. J. Oper. Res..

[15] Poeting M., Schaudt S., Clausen U.A. Comprehensive Case Study in Last-Mile Delivery Concepts for Parcel Robots. Proceedings of the Winter Simulation Conference (WSC).

[16] Abrar M.M., Islam R., Shanto M.A.H. An Autonomous Delivery Robot to Prevent the Spread of Coronavirus in Product Delivery System. Proceedings of the IEEE Annual Ubiquitous Computing, Electronics & Mobile Communication Conference (UEMCON).

[17] Samouh F., Gluza V., Djavadian S., Meshkani S., Farooq B. Multimodal Autonomous Last-Mile Delivery System Design and Application. Proceedings of the IEEE International Smart Cities Conference (ISC2).

[18] Gao F., Cheng Y., Gao M., Ma C., Liu H., Ren Q., Zhao Z. Design and Implementation of an Autonomous Driving Delivery Robot. Proceedings of the Chinese Control Conference (CCC).

[19] Hutter M., Gehring C., Lauber A., Gunther F., Bellicoso C.D., Tsounis V., Meyer K. (2017). Anymal-toward legged robots for harsh environments. Adv. Rob..

[20] Castillo G.A., Weng B., Zhang W., Hereid A. Robust Feedback Motion Policy Design Using Reinforcement Learning on a 3D Digit Bipedal Robot. Proceedings of the IEEE/RSJ International Conference on Intelligent Robots and Systems (IROS).

[21] Galindo C., Fernández-Madrigal J.-A., González J., Saffiotti A. (2008). Robot task planning using semantic maps. Robot. Auton. Syst..

[22] Abed M., Farouq O., Doori Q.A. (2021). A Review on Path Planning Algorithms for Mobile Robots. Eng. Technol. J..

[23] Rafai A.N.A., Adzhar N., Jaini N.I., Ding B. (2022). A Review on Path Planning and Obstacle Avoidance Algorithms for Autonomous Mobile Robots. J. Robot..

[24] Fouque C., Bonnifait P. On the use of 2D navigable maps for enhancing ground vehicle localization. Proceedings of the 2009 IEEE/RSJ International Conference on Intelligent Robots and Systems.

[25] Liu L., Wang X., Yang X., Liu H., Li J., Wang P. (2023). Path planning techniques for mobile robots: Review and prospect. Expert Syst. Appl..

[26] De Ryck M., Versteyhe M., Debrouwere F. (2020). Automated guided vehicle systems, state-of-the-art control algorithms and techniques. J. Manuf. Syst..

[27] Kim J.-T., Choi Y.-H., Lee J., Hong S.-H. Floor-to-floor navigation for a mobile robot. Proceedings of the 2013 10th International Conference on Ubiquitous Robots and Ambient Intelligence (URAI).

[28] Zhang H., Tao W., Huang J., Zheng R. Development of An In-building Transport Robot for Autonomous Usage of Elevators. Proceedings of the 2018 IEEE International Conference on Intelligence and Safety for Robotics (ISR).

[29] Law W.-t., Li K.-s., Fan K.-w., Mo T., Poon C.-k. Friendly Elevator Co-rider: An HRI Approach for Robot-Elevator Interaction. Proceedings of the 17th ACM/IEEE International Conference on Human-Robot Interaction (HRI).

[30] Rubies E., Bitriá R., Clotet E., Palacín J. (2023). Non-Contact and Non-Intrusive Add-on IoT Device for Wireless Remote Elevator Control. Appl. Sci..

[31] Palacín J., Bitriá R., Rubies E., Clotet E. (2023). A Procedure for Taking a Remotely Controlled Elevator with an Autonomous Mobile Robot Based on 2D LIDAR. Sensors.

[32] Arisumi H., Chardonnet J.-R., Yokoi K. Whole-body motion of a humanoid robot for passing through a door—Opening a door by impulsive force. Proceedings of the 2009 IEEE/RSJ International Conference on Intelligent Robots and Systems.

[33] Digioia G., Arisumi H., Yokoi K. Trajectory planner for a humanoid robot passing through a door. Proceedings of the 9th IEEE-RAS International Conference on Humanoid Robots.

[34] Kwak N., Arisumi H., Yokoi K. Visual recognition of a door and its knob for a humanoid robot. Proceedings of the IEEE International Conference on Robotics and Automation.

[35] Arisumi H., Kwak N., Yokoi K. Systematic touch scheme for a humanoid robot to grasp a door knob. Proceedings of the IEEE International Conference on Robotics and Automation.

[36] Banerjee N., Long X., Du R., Polido F., Feng S., Atkeson C.G., Gennert M., Padir T. Human-supervised control of the ATLAS humanoid robot for traversing doors. Proceedings of the IEEE-RAS 15th International Conference on Humanoid Robots (Humanoids).

[37] Thrun S., Lakemeyer G., Nebel B. (2003). Robotic mapping: A survey. Exploring Artificial Intelligence in the New Millenium.

[38] Chen C., Cheng Y. Research on Map Building by Mobile Robots. Proceedings of the 2008 International Symposium on Intelligent Information Technology Application.

[39] Asada M. (1990). Map building for a mobile robot from sensory data. IEEE Trans. Syst. Man Cybern..

[40] Kuipers B., Byun Y.-T. (1991). A robot exploration and mapping strategy based on a semantic hierarchy of spatial representations. Robot. Auton. Syst..

[41] Kröse B.J.A., Vlassis N., Bunschoten R., Motomura Y. (2001). A probabilistic model for appearance-based robot localization. Image Vis. Comput..

[42] Jacky C.H., George L.C.S., Charlie H.Y., Lu Y.-H. Multi-robot SLAM with topological/metric maps. Proceedings of the IEEE/RSJ International Conference on Intelligent Robots and Systems.

[43] Choset H., Nagatani K. (2001). Topological simultaneous localization and mapping (SLAM): Toward exact localization without explicit localization. IEEE Trans. Robot. Autom..

[44] Lee D., Chung W., Kim M. A reliable position estimation method of the service robot by map matching. Proceedings of the IEEE International Conference on Robotics and Automation.

[45] Zhou J.-H., Lin H.-Y. A self-localization and path planning technique for mobile robot navigation. Proceedings of the 9th World Congress on Intelligent Control and Automation.

[46] Kubota N. Topological approaches for simultaneous localization and mapping. Proceedings of the 6th International Conference on Informatics, Electronics and Vision & 2017 International Symposium in Computational Medical and Health Technology.

[47] Warrier A.R., Nedunghat P., Bera M.K., Nath K. Implementation of Classical Path Planning Algorithms for Mobile Robot Navigation: A Comprehensive Comparison. Proceedings of the International Conference on Electrical, Computer, Communications and Mechatronics Engineering.

[48] Sundar K., Misra S., Rathinam S., Sharma R. Routing unmanned vehicles in GPS-denied environments. Proceedings of the International Conference on Unmanned Aircraft Systems (ICUAS).

[49] Žunić E., Hindija H., Beširević A., Hodžić K., Delalić S. Improving Performance of Vehicle Routing Algorithms using GPS Data. Proceedings of the 14th Symposium on Neural Networks and Applications (NEUREL).

[50] Aqel M.O.A., Marhaban M.H., Saripan M.I., Ismail N.B. (2016). Review of visual odometry: Types, approaches, challenges, and applications. SpringerPlus.

[51] Bârsan I.A., Liu P., Pollefeys M., Geiger A. Robust dense mapping for large-scale dynamic environments. Proceedings of the IEEE International Conference on Robotics and Automation.

[52] Ji K., Chen H., Di H., Gong J., Xiong G., Qi J., Yi T. CPFG-SLAM: A robust simultaneous localization and mapping based on LIDAR in off-road environment. Proceedings of the IEEE Intelligent Vehicles Symposium (IV).

[53] Du S., Li Y., Li X., Wu M. (2021). LiDAR Odometry and Mapping Based on Semantic Information for Outdoor Environment. Remote Sens..

[54] Chen Y., Medioni G. Object modeling by registration of multiple range images. Proceedings of the IEEE International Conference on Robotics and Automation.

[55] Besl P.J., McKay N.D. (1992). A Method for Registration of 3-D Shapes. IEEE Trans. Pattern Anal. Mach. Intell..

[56] Yokozuka M., Koide K., Oishi S., Banno A. LiTAMIN: LiDAR-Based Tracking and Mapping by Stabilized ICP for Geometry Approximation with Normal Distributions. Proceedings of the IEEE/RSJ International Conference on Intelligent Robots and Systems (IROS).

[57] Koide K., Miura J., Menegatti E. (2019). A portable three-dimensional LIDAR-based system for long-term and wide-area people behavior measurement. Int. J. Adv. Robot. Syst..

[58] Behley J., Stachniss C. Efficient Surfel-Based SLAM using 3D Laser Range Data in Urban Environments. Proceedings of the International Conference on Robotics: Science and Systems (RSS).

[59] Park C., Moghadam P., Kim S., Elfes A., Fookes C., Sridharan S. Elastic LiDAR Fusion: Dense Map-Centric Continuous-Time SLAM. Proceedings of the International Conference on Robotics and Automation (ICRA).

[60] Whelan T., Leutenegger S., Moreno R.F.S., Glocker B., Davison A.J. ElasticFusion: Dense SLAM without a Pose Graph. Proceedings of the International Conference of Robotics: Science and Systems (RSS).

[61] Moosmann F., Stiller C. Velodyne SLAM. Proceedings of the 2011 IEEE Intelligent Vehicles Symposium (IV).

[62] Droeschel D., Behnke S. Efficient Continuous-time SLAM for 3D Lidar-based Online Mapping. Proceedings of the International Conference on Robotics and Automation (ICRA).

[63] Palacín J., Martínez D., Rubies E., Clotet E. (2020). Mobile Robot Self-Localization with 2D Push-Broom LIDAR in a 2D Map. Sensors.

[64] Zhang J., Singh S. LOAM: Lidar Odometry and Mapping in Real-time. Proceedings of the International Conference of Robotics: Science and Systems (RSS).

[65] Zhang J., Singh S. (2017). Low-drift and Real-time Lidar Odometry and Mapping. Auton. Robot..

[66] Shan T., Englot B. LeGO-LOAM: Lightweight and Ground- Optimized Lidar Odometry and Mapping on Variable Terrain. Proceedings of the IEEE/RSJ International Conference on Intelligent Robots and Systems (IROS).

[67] Ye H., Chen Y., Liu M. Tightly Coupled 3D Lidar Inertial Odometry and Mapping. Proceedings of the International Conference on Robotics and Automation.

[68] Shan T., Englot B., Meyers D., Wang W., Ratti C., Rus D. LIO-SAM: Tightly-coupled Lidar Inertial Odometry via Smoothing and Mapping. Proceedings of the IEEE/RSJ International Conference on Intelligent Robots and Systems (IROS).

[69] Qin C., Ye H., Pranata C.E., Han J., Zhang S., Liu M. LINS: A Lidar-Inertial State Estimator for Robust and Efficient Navigation. Proceedings of the IEEE International Conference on Robotics and Automation (ICRA).

[70] LeCun Y., Bengio Y., Hinton G. (2015). Deep Learning. Nature.

[71] Zheng C., Lyu Y., Li M., Zhang Z. Lodonet: A deep neural network with 2D keypoint matching for 3d lidar odometry estimation. Proceedings of the ACM International Conference on Multimedia.

[72] Li Z., Wang N. Dmlo: Deep matching lidar odometry. Proceedings of the IEEE/RSJ International Conference on Intelligent Robots and Systems (IROS).

[73] Li Q., Chen S., Wang C., Li X., Wen C., Cheng M., Li J. LO-net: Deep real-time lidar odometry. Proceedings of the IEEE/CVF Conference on Computer Vision and Pattern Recognition.

[74] Nubert J., Khattak S., Hutter M. Self-supervised learning of lidar odometry for robotic applications. Proceedings of the IEEE International Conference on Robotics and Automation (ICRA).

[75] Wang M., Saputra M.R.U., Zhao P., Gusmao P., Yang B., Chen C., Markham A., Trigoni N. Deeppco: End-to-end point cloud odometry through deep parallel neural network. Proceedings of the EEE/RSJ International Conference on Intelligent Robots and Systems (IROS).

[76] Cho Y., Kim G., Kim A. Unsupervised geometry-aware deep lidar odometry. Proceedings of the IEEE International Conference on Robotics and Automation (ICRA).

[77] Wang G., Wu X., Liu Z., Wang H. Pwclo-net: Deep lidar odometry in 3d point clouds using hierarchical embedding mask optimization. Proceedings of the IEEE/CVF Conference on Computer Vision and Pattern Recognition (CVPR).

[78] Wang C., Liu X., Yang X., Hu F., Jiang A., Yang C. (2018). Trajectory Tracking of an Omni-Directional Wheeled Mobile Robot Using a Model Predictive Control Strategy. Appl. Sci..

[79] Palacín J., Rubies E., Clotet E., Martínez D. (2021). Evaluation of the Path-Tracking Accuracy of a Three-Wheeled Omnidirectional Mobile Robot Designed as a Personal Assistant. Sensors.

[80] Kang J.G., An S.Y., Oh S.Y. Navigation strategy for the service robot in the elevator environment. Proceedings of the International Conference on Control, Automation and Systems.

[81] van Toll W., Cook A.F., Geraerts R. Navigation meshes for realistic multi-layered environments. Proceedings of the IEEE/RSJ International Conference on Intelligent Robots and Systems.

[82] Zhang Q., Wu X., Liu B., Adiwahono A.H., Dung T.A., Chang T.W. A hierarchical topological planner for multi-storey building navigation. Proceedings of the IEEE International Conference on Advanced Intelligent Mechatronics (AIM).

[83] Liu K., Motta G., Ma T., Guo T. Multi-floor Indoor Navigation with Geomagnetic Field Positioning and Ant Colony Optimization Algorithm. Proceedings of the IEEE Symposium on Service-Oriented System Engineering (SOSE).

[84] Joo S.H., Manzoor S., Kuc T.Y. A Semantic Navigation Framework for Multi-Floor Building Environment. Proceedings of the International Conference on Control, Automation and Systems (ICCAS).

[85] Li Z. Using MDP to Find the Best Path in Multi-floor World. Proceedings of the IEEE International Conference on Frontiers Technology of Information and Computer (ICFTIC).

[86] Yuan J., Jiao B., Wang L. Indoor and outdoor integrated path planning algorithm for multi-storey buildings. Proceedings of the 2022 World Automation Congress (WAC).

[87] Fransen K., van Eekelen J. (2023). Efficient path planning for automated guided vehicles using A* (Astar) algorithm incorporating turning costs in search heuristic. Int. J. Prod. Res..

[88] Dijkstra E.W. (1959). A note on two problems in connexion with graphs. Numer. Math..

[89] Floyd R.W. (1962). Algorithm 97: Shortest Path. Commun. ACM.

[90] Hart P.E., Nilsson N.J., Raphael B. (1968). A Formal Basis for the Heuristic Determination of Minimum Cost Paths. IEEE Trans. Syst. Sci. Cybern..

[91] Holland J.H., Selfridge O.G., Rissland E.L., Arbib M.A. (1984). Genetic Algorithms and Adaptation. Adaptive Control of Ill-Defined Systems.

[92] Kirkpatrick S., Gelatt C.D., Vecchi M.P. (1983). Optimization by Simulated Annealing. Science.

[93] Lawler E.L., Wood D.E. (1966). Branch-And-Bound Methods: A Survey. Oper. Res..

[94] Griffiths I.J., Mehdi Q.H., Wang T., Gough N.E. (1997). A Genetic Algorithm for Path Planning. IFAC Proc. Vol..

[95] Lamini C., Benhlima S., Elbekri A. (2018). Genetic Algorithm Based Approach for Autonomous Mobile Robot Path Planning. Procedia Comput. Sci..

[96] Kusuma M., Riyanto, Machbub C. Humanoid Robot Path Planning and Rerouting Using A-Star Search Algorithm. Proceedings of the IEEE International Conference on Signals and Systems.

[97] Ganeshmurthy M.S., Suresh G.R. Path planning algorithm for autonomous mobile robot in dynamic environment. Proceedings of the 3rd International Conference on Signal Processing, Communication and Networking.

[98] Tsuzuki M.S.G., Martins T.C., Takase F.K. (2006). Robot path planning using simulated annealing. IFAC Proc. Vol..

[99] Miao H., Tian Y.-C. Robot path planning in dynamic environments using a simulated annealing based approach. Proceedings of the 10th International Conference on Control, Automation, Robotics and Vision.

[100] Kim J., Jung H. (2022). Robot Routing Problem of Last-Mile Delivery in Indoor Environments. Appl. Sci..

[101] Palacín J., Rubies E., Clotet E. (2022). The Assistant Personal Robot Project: From the APR-01 to the APR-02 Mobile Robot Prototypes. Designs.

[102] Palacín J., Martínez D., Rubies E., Clotet E. (2021). Suboptimal Omnidirectional Wheel Design and Implementation. Sensors.

[103] Palacín J., Clotet E., Martínez D., Martínez D., Moreno J. (2019). Extending the Application of an Assistant Personal Robot as a Walk-Helper Tool. Robotics.

[104] Mori Y., Yokoyama S., Yamashita T., Kawamura H., Mori M. Obstacle Avoidance Using Depth Imaging for Forearm-Supported Four-Wheeled Walker with Walking Assist. Proceedings of the International Conference on Ubiquitous Robots (UR).

[105] Tian P., Zhang Y.N., Zhang J., Yan N.M., Zeng W. (2013). Research on Simulation of Motion Compensation for 8×8 Omnidirectional Platform Based on Back Propagation Network. Appl. Mech. Mater..

[106] Peng T., Qian J., Zi B., Liu J., Wang X. (2016). Mechanical Design and Control System of an Omni-directional Mobile Robot for Material Conveying. Procedia CIRP.

[107] Wang Z., Yang G., Su X., Schwager M., Groß R., Kolling A., Berman S., Frazzoli E., Martinoli A., Matsuno F., Gauci M. (2018). OuijaBots: Omnidirectional Robots for Cooperative Object Transport with Rotation Control Using No Communication. Distributed Autonomous Robotic Systems.

[108] Li Y., Ge S., Dai S., Zhao L., Yan X., Zheng Y., Shi Y. (2020). Kinematic Modeling of a Combined System of Multiple Mecanum-Wheeled Robots with Velocity Compensation. Sensors.

[109] Purwin O., D’Andrea R. (2006). Trajectory generation and control for four wheeled omnidirectional vehicles. Robot. Auton. Syst..

[110] Kim K.B., Kim B.K. (2011). Minimum-Time Trajectory for Three-Wheeled Omnidirectional Mobile Robots Following a Bounded-Curvature Path with a Referenced Heading Profile. IEEE Trans. Robot..

[111] Jia W., Zhao W., Song Z., Li Z. Object Servoing of Differential-Drive Robots. Proceedings of the Chinese Control and Decision Conference (CCDC).

[112] Bitriá R., Palacín J. (2022). Optimal PID Control of a Brushed DC Motor with an Embedded Low-Cost Magnetic Quadrature Encoder for Improved Step Overshoot and Undershoot Responses in a Mobile Robot Application. Sensors.

[113] Palacín J., Rubies E., Clotet E. (2022). Systematic Odometry Error Evaluation and Correction in a Human-Sized Three-Wheeled Omnidirectional Mobile Robot Using Flower-Shaped Calibration Trajectories. Appl. Sci..

[114] Clotet E., Palacín J. (2023). SLAMICP Library: Accelerating Obstacle Detection in Mobile Robot Navigation via Outlier Monitoring following ICP Localization. Sensors.

[115] Styan G.P.H. (1973). Hadamard products and multivariate statistical analysis. Linear Algebra Its Appl..

[116] Makariye N. Towards shortest path computation using Dijkstra algorithm. Proceedings of the 2017 International Conference on IoT and Application (ICIOT).

[117] Asadi S., Azimirad V., Eslami A., Ghanbari A. A novel global optimal path planning and trajectory method based on adaptive dijkstra-immune approach for mobile robot. Proceedings of the IEEE/ASME International Conference on Advanced Intelligent Mechatronics (AIM).

[118] Fusic J.S., Ramkumar P., Hariharan K. Path planning of robot using modified Dijkstra Algorithm. Proceedings of the 2018 National Power Engineering Conference (NPEC).

[119] Wang H., Yu Y., Yuan Q. Application of Dijkstra algorithm in robot path-planning. Proceedings of the International Conference on Mechanic Automation and Control Engineering.

[120] Li X. (2021). Path planning of intelligent mobile robot based on Dijkstra algorithm. J. Phys. Conf. Ser..

[121] Alshammrei S., Boubaker S., Kolsi L. (2022). Improved Dijkstra algorithm for mobile robot path planning and obstacle avoidance. Comput. Mater. Contin..

[122] Dijkstra’s Shortest Path Algorithm Explained. https://youtu.be/bZkzH5x0SKU.

[123] Dijkstra’s Algorithm MATLAB Central File Exchange. https://www.mathworks.com/matlabcentral/fileexchange/134851-dijkstra.

[124] Khatib O. Real-time obstacle avoidance for manipulators and mobile robots. Proceedings of the IEEE International Conference on Robotics and Automation.

[125] Zhang Y., Liu K., Gao F., Zhao F. (2023). Research on Path Planning and Path Tracking Control of Autonomous Vehicles Based on Improved APF and SMC. Sensors.

[126] Hough D.G. (2019). The IEEE Standard 754: One for the History Books. Computer.

[127] Kunchev V., Jain L., Ivancevic V., Finn A., Gabrys B., Howlett R.J., Jain L.C. (2006). Path Planning and Obstacle Avoidance for Autonomous Mobile Robots: A Review. Knowledge-Based Intelligent Information and Engineering Systems.

[128] Anavatti S.G., Francis S.L.X., Garratt M. Path-planning modules for Autonomous Vehicles: Current status and challenges. Proceedings of the International Conference on Advanced Mechatronics, Intelligent Manufacture, and Industrial Automation (ICAMIMIA).

